# Investigating tralokinumab-related adverse events in treating atopic dermatitis: insights from the FAERS database

**DOI:** 10.3389/fimmu.2026.1769109

**Published:** 2026-02-23

**Authors:** Yueping Jiang, Xinjuan Sun, Yang Li

**Affiliations:** 1 Department of Pharmacy, Gaochun Traditional Chinese Medicine Hospital, Nanjing, Jiangsu, China; 2 Department of Endocrinology, Air Force Hospital of Eastern Theater Command, Nanjing, Jiangsu, China; 3 Department of Pharmacy, Nanjing First Hospital, Nanjing Medical University, Nanjing, Jiangsu, China

**Keywords:** conjunctivitis, dupilumab, FAERS, injection site reactions, keratitis, pharmacovigilance, tralokinumab

## Abstract

**Objectives:**

Dupilumab and tralokinumab are FDA-approved biological agents for the treatment of atopic dermatitis (AD). This study analyzed tralokinumab-related adverse events (AEs) reported by healthcare professionals, utilizing data mined from the FDA Adverse Event Reporting System (FAERS). Furthermore, we compared the frequency of reports for common AEs with dupilumab or tralokinumab as the primary suspect, focusing on injection-site reactions, conjunctivitis, and keratitis.

**Methods:**

Disproportionality analysis methods, including the reporting odds ratio (ROR), the Medicines and Healthcare products Regulatory Agency (MHRA) comprehensive method, the Bayesian confidence propagation neural network (BCPNN), and the Multi-item gamma Poisson shrinker (MGPS), were employed to quantify tralokinumab-associated AE signals. Then, the occurrence risk of common AEs between dupilumab and tralokinumab was further compared.

**Results:**

Among 1,591,367 AE reports, 1,770 identified tralokinumab as the primary suspect. Tralokinumab-related AEs affected 25 System Organ Classes (SOC), with 49 significant disproportionality primary terms (PTs) consistently detected across all four algorithms. Notable potential AEs included eczema herpeticum, generalized exfoliative dermatitis, alopecia, skin exfoliation, and blepharitis. Most tralokinumab-related AEs occurred within the first month of treatment, with a median onset time of 37 days (interquartile range [IQR]: 13–111 days). The reporting proportion of injection site reactions was significantly higher with dupilumab than with tralokinumab (p < 0.001). In contrast, tralokinumab was associated with a substantially higher reporting proportion of conjunctivitis (p = 0.001) and keratitis (p = 0.039).

**Conclusion:**

This study identified potential AE signals that could aid clinical monitoring and risk identification for tralokinumab. Additionally, close monitoring is warranted for dupilumab-associated injection site reactions and tralokinumab-associated conjunctivitis and keratitis throughout treatment.

## Introduction

AD is an inflammatory skin condition with diverse manifestations stemming from interactions between genetic predispositions and environmental triggers that compromise skin barrier integrity and immune function (1, 2). Severe itching is a common symptom that markedly impairs a patient’s quality of life. The incidence rates of AD in adults and children in different countries and regions are 3.4%-33.7% and 13.5%-41.9%, respectively (3, 4). Targeted treatments are being developed to improve long-term therapies for patients with moderate-to-severe AD owing to a better understanding of the inflammatory pathways of the disease. Recent studies have identified interleukin-13 (IL-13), a type 2 cytokine, as a crucial factor in AD-related inflammation (5, 6). IL-13 is associated with breakdown of the skin barrier, skin inflammation, increased risk of skin infections, itch signaling, and excessive epidermal growth, with IL-13 levels in the affected skin correlating with the severity of AD (7).

Tralokinumab, a fully human IgG4 monoclonal antibody, binds strongly and exclusively to IL-13, blocking its connection with the receptor and the resulting signaling pathways (8). Tralokinumab demonstrated notable and prompt improvements in the scope and severity of AD in clinical trials involving moderate-to-severe cases, with a safety profile similar to placebo throughout the 52 weeks (9–11). Consequently, they are commonly used in the clinical setting. The most frequent AEs observed with tralokinumab compared with placebo included viral upper respiratory tract infection, upper respiratory tract infection, conjunctivitis, and injection-site reactions (11). Currently, safety data on tralokinumab are scarce, particularly from large-scale studies and real-world experience. Therefore, it is recommended that post-marketing surveillance of tralokinumab be conducted using data-mining algorithms that comprehensively scan large datasets to detect potential AE signals.

Dupilumab, another approved human monoclonal IgG4 antibody, is indicated for the treatment of moderate-to-severe AD and asthma. Dupilumab inhibits the IL-4 and IL-13 signaling pathways by binding to the IL-4 receptor alpha subunit (IL-4Ra), which is shared by the IL-4 and IL-13 receptors, thereby impeding receptor activation and subsequent signaling. Consequently, there is a reduction in epidermal proliferation markers and inflammatory mediators, alongside an increase in structural proteins, lipid metabolism proteins, and epidermal barrier proteins, thereby restoring normal skin function.

In this study, we conducted a retrospective analysis of tralokinumab-associated AEs based on the FAERS database. Additionally, our study compared the proportions of injection-site reactions, conjunctivitis, and keratitis reported with tralokinumab and dupilumab.

## Methods

### Data source and deduplication

We conducted a retrospective pharmacovigilance study analyzing AE data from the FAERS database for the period Q4–2021 to Q2 2025. The FAERS database collects AE reports from a variety of sources, including health professionals, pharmaceutical companies, consumers, and individuals outside the healthcare industry, through spontaneous reporting. Seven datasets categorized FAERS data: demographic and administrative details (DEMO), drug specifics (DRUG), adverse drug reaction details (REAC), patient outcomes (OUTC), sources of reports (RPSR), drug therapy start and end dates (THER), and drug indication information (INDI). Our study adhered to FDA guidelines for data analysis by selecting the most recent entry for each case, based on the latest FDA_DT, when duplicate CASEIDs were present. In addition, if both CASEID and FDA_DT are the same, the entry with the greater PRIMARYID value is selected. This process ensured the exclusion of duplicate data entries, allowing for more accurate assessment of AEs associated with tralokinumab.

Following deduplication, we further implemented targeted data cleaning for key variables to address incomplete or implausible records: For age, records with missing values were retained to support descriptive demographic analysis, while implausible entries (e.g., age < 0 or age > 120) were identified and excluded to maintain data validity.

Onset time was calculated as the interval between EVENT_DT (adverse event date) and START_DT (tralokinumab initiation date). Reports containing temporal inconsistencies (e.g., EVENT_DT preceding START_DT) or missing medication start/end times were excluded from the analysis. The ROR method was utilized to identify signals of common AEs associated with tralokinumab and dupilumab. AE signals related to the two drugs were compared from each drug’s market authorization through Q2 2025.

### AEs and drug detection

AEs documented in the FAERS database were categorized according to the Medical Dictionary for Regulatory Activities (MedDRA) Information Classification version 27.1. In our study, we identified and analyzed all AEs associated with tralokinumab by extracting data from the ‘REAC’ files within the FAERS database. The analysis focused on evaluating the proportion and severity of AEs using MedDRA SOC and PT categories.

In our study, the target drugs were identified using their generic name, tralokinumab, and the brand name, Adbry, in the DRUG dictionary. To enhance accuracy, we confined our analysis to reports where the drug’s role code was designated as primary suspect (PS) in the DRUG files. To improve the precision of our data analysis, we focused exclusively on adverse reaction reports submitted by medical professionals, identified using the “OCCP_CODE” field in the FAERS database. This field contains codes indicating the reporter’s occupation, allowing us to filter reports submitted by physicians, nurses, pharmacists, and other healthcare professionals.

### Data mining algorithm

Disproportionality analysis was used to evaluate the possible association between tralokinumab and AEs. The study of disproportionality is considered a crucial method in pharmacovigilance research, using four algorithms: the reporting odds ratio (ROR), the Medicines and Healthcare products Regulatory Agency (MHRA) comprehensive, the Bayesian confidence propagation neural network (BCPNN), and the multi-item gamma Poisson shrinker (MGPS). The equations and criteria for the four algorithms have been previously described and are displayed in **Supplementary Table S1**. One of the four algorithms met these requirements and should be viewed as a promising indicator of drug-associated AEs. For this study, we selected AE signals that satisfied the requirements of the four algorithms discussed earlier. Each obtained AE signal was compared against the drug package insert. If not mentioned in the package insert, the signal was considered an unexpected or novel AE signal.

### Statistical analysis

**Figure 1:**
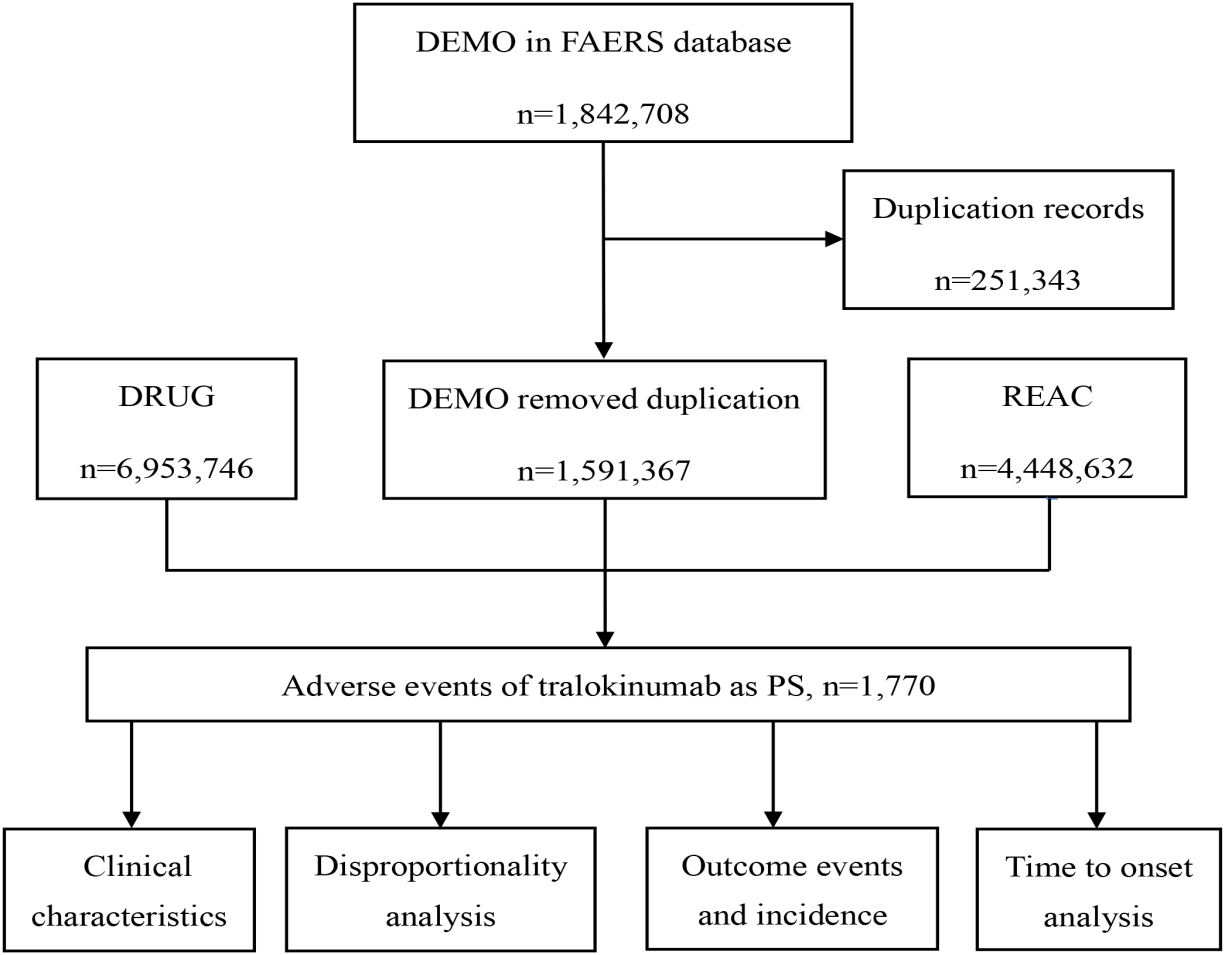
Flowchart of adverse events screening for tralokinumab in the FAERS database.

To further compare the safety profiles of tralokinumab and dupilumab, the chi-square test was used to assess differences in the proportions of selected common AEs, including injection site reactions, conjunctivitis, and keratitis. A p-value of < 0.05 was considered statistically significant. **Figure 1** shows the flow chart of our research. RStudio 4.3.2, SPSS 27.0, and Microsoft Excel 2021 were used for all data processing and statistical analyses.

### Limitations of the study

This study has several significant limitations that must be emphasized to ensure proper interpretation of the results. These limitations primarily stem from the inherent characteristics of the FAERS database and restrictions on data completeness.

First, FAERS data cannot establish causality; they can only show statistical associations. The pharmacovigilance signals detected in this analysis reflect statistical correlations rather than definitive causal relationships. Second, reporting in FAERS is spontaneous, leading to under-reporting, reporting biases, and the inability to calculate incidence rates. The database captures only the relative frequency of AE reports and lacks incidence or prevalence data from real-world clinical settings. Consequently, associations derived from disproportionality metrics should not be directly extrapolated to clinical incidence rates and should be further validated using complementary data sources. Third, confounding by indication, concomitant medications, and underlying disease severity cannot be controlled for. This severely limits the ability to attribute AEs directly to tralokinumab versus the natural history of severe AD or other treatments. Clinical decision-making should take into account the potential impact of these confounding factors.

## Results

### Descriptive analysis

From Q4 2021 to Q2 2025, 1,591,367 entries were documented in the FAERS system, with 1,770 reports listing tralokinumab as the PS and 3,137 associated AEs. **Table 1** summarizes the clinical characteristics of tralokinumab-associated events. Females accounted for 62.5% of AE reports, with the majority originating in the United States (93.2%). AD was the most common indication (67.8%), which partially diverged from the labeled indications. Serious outcomes included hospitalization (4.1% of reports), death (1.1% of reports), and life-threatening events (0.3% of reports).

**Table 1:** Characteristics of reports associated with tralokinumab from the FAERS database.

Characteristics	Case number, n	Case proportion, %
Number of events	1770	
Gender		
Male	656	37.1
Female	1106	62.5
Missing	8	0.5
Age		
<18	59	3.3
≥18,<65	1124	63.5
≥65	273	15.4
Missing	314	17.7
Outcome of AEs		
Hospitalization	72	4.1
Death	19	1.1
Life-threatening	5	0.3
Other serious events	1674	94.5
Reported countries		
USA	1649	93.2
Others	121	6.8
Reported person		
Physician	212	12.0
Pharmacist	1558	88.0
Reporting year		
2022	151	8.5
2023	350	19.8
2024	643	36.3
2025Q1-Q2	626	35.4

### Signal detection

**Table 2** presents the signal strength and frequency of AEs associated with tralokinumab across different SOCs. While reports covered 25 SOCs, only four showed significant safety signals meeting the criteria for at least one detection method: skin and subcutaneous tissue disorders, general disorders and administration site conditions, eye disorders, and immune system disorders.

**Table 2:** Signal strength of reports of tralokinumab at the system organ class level in the FAERS database.

System organ class (SOC)	Case reports	ROR (95% CI)	MHRA (χ2)	EBGM (EBGM05)	IC (IC025)
Skin and subcutaneous tissue disorders*	1141	**7.71 (7.17 - 8.29)**	**5.27 (4222.59)**	**5.25 (4.88)**	**2.39 (2.29)**
General disorders and administration site conditions*	751	**1.85 (1.7 - 2.01)**	1.65 (222.61)	1.65 (1.52)	**0.72 (0.6)**
Eye disorders*	357	**5.6 (5.01 - 6.25)**	**5.07 (1189.89)**	**5.06 (4.53)**	**2.34 (2.16)**
Infections and infestations	144	0.72 (0.61 - 0.85)	0.73 (14.77)	0.73 (0.62)	-0.45 (-0.69)
Injury, poisoning, and procedural complications	130	0.33 (0.28 - 0.39)	0.36 (170.87)	0.36 (0.3)	-1.49 (-1.74)
Nervous system disorders	122	0.57 (0.47 - 0.68)	0.58 (38.86)	0.58 (0.49)	-0.78 (-1.04)
Musculoskeletal and connective tissue disorders	88	0.7 (0.57 - 0.87)	0.71 (10.83)	0.71 (0.57)	-0.49 (-0.8)
Gastrointestinal disorders	71	0.3 (0.23 - 0.37)	0.31 (116.12)	0.31 (0.25)	-1.68 (-2.01)
Respiratory, thoracic, and mediastinal disorders	60	0.37 (0.29 - 0.48)	0.39 (61.35)	0.39 (0.3)	-1.37 (-1.73)
Immune system disorders*	59	**1.43 (1.11 - 1.86)**	1.43 (7.62)	1.43 (1.1)	**0.51 (0.13)**
Psychiatric disorders	42	0.31 (0.23 - 0.43)	0.32 (61.94)	0.32 (0.24)	-1.63 (-2.05)
Investigations	41	0.21 (0.16 - 0.29)	0.22 (117.86)	0.22 (0.16)	-2.16 (-2.59)
Surgical and medical procedures	24	0.69 (0.46 - 1.03)	0.69 (3.37)	0.69 (0.46)	-0.53 (-1.1)
Neoplasms benign, malignant, and unspecified (incl cysts and polyps)	19	0.11 (0.07 - 0.16)	0.11 (143.95)	0.11 (0.07)	-3.18 (-3.76)
Vascular disorders	18	0.29 (0.18 - 0.45)	0.29 (32.08)	0.29 (0.18)	-1.79 (-2.4)
Reproductive system and breast disorders	14	1 (0.59 - 1.69)	1 (0)	1 (0.59)	0 (-0.75)
Metabolism and nutrition disorders	11	0.14 (0.08 - 0.25)	0.14 (59.75)	0.14 (0.08)	-2.84 (-3.57)
Renal and urinary disorders	10	0.15 (0.08 - 0.29)	0.16 (46.66)	0.16 (0.08)	-2.68 (-3.44)
Cardiac disorders	8	0.1 (0.05 - 0.19)	0.1 (68.65)	0.1 (0.05)	-3.36 (-4.17)
Blood and lymphatic system disorders	8	0.08 (0.04 - 0.15)	0.08 (90.19)	0.08 (0.04)	-3.68 (-4.49)
Product issues	6	0.14 (0.06 - 0.31)	0.14 (31.5)	0.14 (0.06)	-2.81 (-3.72)
Ear and labyrinth disorders	6	0.66 (0.3 - 1.48)	0.66 (1.03)	0.66 (0.3)	-0.59 (-1.61)
Endocrine disorders	3	0.19 (0.06 - 0.59)	0.19 (10.24)	0.19 (0.06)	-2.38 (-3.5)
Hepatobiliary disorders	2	0.04 (0.01 - 0.16)	0.04 (47.06)	0.04 (0.01)	-4.65 (-5.76)
Congenital, familial, and genetic disorders	2	0.2 (0.05 - 0.8)	0.2 (6.4)	0.2 (0.05)	-2.32 (-3.54)

Bold values indicate system organ classes that meet all four signal calculation methods simultaneously.

**Table 3** identifies 49 tralokinumab-related AEs across five SOCs, including labeled events (e.g., conjunctivitis, injection site erythema, and swelling) and unexpected findings (e.g., eczema herpeticum, generalized dermatitis exfoliative, alopecia, skin exfoliation, and blepharitis). Although listed on the drug label, upper respiratory tract infections did not satisfy the criteria for any of the four algorithms assessed. As angioedema and eosinophilia were reported in fewer than three cases, they were not included in the study outcomes. Given that the FAERS database captures all medically significant and health-related PTs, our analysis also uncovered signals associated with diseases, including pruritus, dry skin, and atopic dermatitis.

**Table 3:** Signal strength of reports of tralokinumab at the Preferred Term level in the FAERS database.

Preferred terms (PTs)	Cases reports	ROR (95% CI)	MHRA (χ^2^)	EBGM (EBGM05)	IC (IC025)
rash	110	3.79 (3.13 - 4.58)	3.69 (217.08)	3.68 (3.04)	1.88 (1.57)
erythema	68	6.33 (4.98 - 8.06)	6.22 (297.49)	6.2 (4.87)	2.63 (2.17)
injection site pain	60	4.33 (3.36 - 5.6)	4.27 (150.46)	4.26 (3.3)	2.09 (1.64)
injection site erythema	47	9.67 (7.25 - 12.92)	9.54 (357.63)	9.49 (7.11)	3.25 (2.59)
injection site swelling	45	9.25 (6.88 - 12.42)	9.13 (324.13)	9.08 (6.76)	3.18 (2.52)
injection site pruritus	43	15.39 (11.37 - 20.83)	15.2 (564.72)	15.05 (11.12)	3.91 (3.07)
eye pruritus	42	12.08 (8.89 - 16.39)	11.93 (417.44)	11.84 (8.72)	3.57 (2.8)
Ocular hyperemia	41	12.44 (9.13 - 16.95)	12.29 (421.98)	12.19 (8.95)	3.61 (2.82)
dry eye	40	7.46 (5.46 - 10.2)	7.38 (219.93)	7.35 (5.38)	2.88 (2.21)
conjunctivitis	38	16.7 (12.1 - 23.04)	16.51 (547.65)	16.33 (11.84)	4.03 (3.08)
vision blurred	32	6.13 (4.32 - 8.69)	6.07 (135.31)	6.05 (4.27)	2.6 (1.89)
**skin exfoliation**	31	4.79 (3.36 - 6.83)	4.75 (91.78)	4.74 (3.33)	2.25 (1.57)
eye irritation	31	10.88 (7.63 - 15.52)	10.78 (273.25)	10.71 (7.51)	3.42 (2.52)
urticaria	27	3.35 (2.29 - 4.9)	3.33 (44.09)	3.33 (2.28)	1.73 (1.07)
hypersensitivity	24	3.16 (2.11 - 4.72)	3.14 (34.99)	3.13 (2.1)	1.65 (0.95)
injection site reaction	24	8.45 (5.65 - 12.64)	8.39 (155.43)	8.35 (5.58)	3.06 (2.11)
**alopecia**	21	3.41 (2.22 - 5.24)	3.4 (35.49)	3.39 (2.21)	1.76 (0.99)
rash macular	19	8.17 (5.2 - 12.85)	8.13 (118.21)	8.09 (5.15)	3.02 (1.93)
eye pain	17	7.9 (4.9 - 12.75)	7.87 (101.38)	7.83 (4.85)	2.97 (1.82)
injection site rash	16	7.93 (4.85 - 12.98)	7.9 (95.91)	7.86 (4.8)	2.97 (1.78)
injection site bruising	14	5.8 (3.43 - 9.82)	5.78 (55.17)	5.76 (3.4)	2.53 (1.38)
lacrimation increased	14	8.21 (4.85 - 13.9)	8.18 (87.74)	8.14 (4.81)	3.02 (1.71)
eye swelling	12	8.12 (4.6 - 14.33)	8.09 (74.18)	8.05 (4.56)	3.01 (1.58)
skin burning sensation	12	5.8 (3.29 - 10.24)	5.78 (47.32)	5.76 (3.27)	2.53 (1.27)
skin fissures	11	3.96 (2.19 - 7.16)	3.95 (24.14)	3.94 (2.18)	1.98 (0.82)
swelling face	11	4.55 (2.51 - 8.23)	4.53 (30.23)	4.52 (2.5)	2.18 (0.97)
ocular discomfort	9	16.57 (8.58 - 31.99)	16.52 (129.74)	16.34 (8.46)	4.03 (1.77)
injection site mass	9	7.04 (3.66 - 13.57)	7.03 (46.31)	7 (3.63)	2.81 (1.21)
injection site warmth	8	14.37 (7.15 - 28.85)	14.33 (98.24)	14.2 (7.07)	3.83 (1.56)
acne	7	4.65 (2.21 - 9.77)	4.64 (19.93)	4.63 (2.2)	2.21 (0.65)
**blepharitis**	6	13.69 (6.12 - 30.62)	13.67 (69.8)	13.55 (6.06)	3.76 (1.18)
swelling of the eyelid	6	9.29 (4.16 - 20.74)	9.27 (44)	9.22 (4.13)	3.2 (0.99)
periorbital swelling	6	10.15 (4.54 - 22.68)	10.13 (49.06)	10.07 (4.51)	3.33 (1.04)
eyelid irritation	6	38.2 (16.97 - 86.01)	38.13 (211.26)	37.16 (16.5)	5.22 (1.48)
injection site haemorrhage	6	5.04 (2.26 - 11.25)	5.04 (19.35)	5.02 (2.25)	2.33 (0.58)
eye inflammation	5	5.24 (2.18 - 12.62)	5.23 (17.06)	5.22 (2.17)	2.38 (0.43)
**eczema herpeticum**	5	56.77 (23.21 - 138.89)	56.68 (263)	54.54 (22.3)	5.77 (1.25)
skin mass	4	10.93 (4.09 - 29.26)	10.92 (35.78)	10.85 (4.05)	3.44 (0.57)
keratitis	4	13.02 (4.86 - 34.86)	13 (43.91)	12.89 (4.81)	3.69 (0.63)
eyelids pruritus	4	12.53 (4.68 - 33.55)	12.51 (42.01)	12.41 (4.64)	3.63 (0.62)
eye discharge	4	5.5 (2.06 - 14.7)	5.5 (14.67)	5.48 (2.05)	2.45 (0.24)
erythema of the eyelid	4	9.85 (3.68 - 26.36)	9.84 (31.56)	9.78 (3.66)	3.29 (0.53)
pigmentation disorder	4	13.58 (5.07 - 36.37)	13.56 (46.1)	13.44 (5.02)	3.75 (0.65)
conjunctivitis allergic	4	25.22 (9.38 - 67.84)	25.19 (91.31)	24.77 (9.21)	4.63 (0.8)
**dermatitis exfoliative generalized**	4	6 (2.25 - 16.04)	6 (16.59)	5.98 (2.24)	2.58 (0.29)
injection site inflammation	4	15.22 (5.68 - 40.78)	15.2 (52.49)	15.05 (5.61)	3.91 (0.68)
xerophthalmia	3	36.68 (11.65 - 115.47)	36.65 (101.41)	35.75 (11.36)	5.16 (0.42)
eye symptom	3	58.29 (18.36 - 185.05)	58.24 (162.11)	55.98 (17.63)	5.81 (0.44)
eyelid rash	3	21.6 (6.9 - 67.59)	21.58 (58)	21.27 (6.8)	4.41 (0.35)

Bold PTs represent AEs not documented in the prescribing information for tralokinumab.

Notably, tralokinumab had a lower reporting proportion of injection-site reactions than dupilumab (15.6% vs. 21.3%, p<0.001), but a higher reporting proportion of conjunctivitis (2.4%vs.1.4%, p=0.001) and keratitis (0.2%vs.0.07%, p=0.039).

### Time-to-onset of AEs

among 97 aes with recorded onset times, the median time to onset was 37 days (interquartile range [iqr]: 13–111 days). by onset time distribution, the highest reporting proportion was observed within the first three months of treatment: 42.3% at 0–30 days, 16.5% at 31–60 days, and 12.4% at 61–90 days. notably, aes with an onset time of more than six months (>180 days) accounted for approximately 14.4%.

## Discussion

This study analyzed pharmacovigilance reports of AEs following tralokinumab approval and evaluated the risk of common AEs, including injection site reactions, conjunctivitis, and keratitis, associated with tralokinumab compared with dupilumab. Tralokinumab-related AEs were more prevalent in women than in men (62.5% vs 37.1%), aligning with epidemiological data showing higher AD prevalence in women than in men (12–14). Estradiol and other female hormones may exacerbate Th2 inflammation in patients with AD (15). The analysis, by SOC, identified the most frequent and clinically significant AEs, including skin and subcutaneous tissue disorders, eye disorders, general disorders and administration site conditions, and immune system disorders. This finding aligns with safety information in the drug label and clinical trials (16, 17).

AEs related to skin and subcutaneous tissue diseases include rash, erythema, skin burning sensation, and alopecia. Notably, alopecia is not included in the drug label. Alopecia areata is a type 1 autoimmune hair loss disorder, and studies have shown increased Th2-related gene expression in skin biopsies from patients with alopecia, suggesting that dupilumab or tralokinumab may be effective treatments. Research reports indicate that patients receiving tralokinumab or dupilumab may experience worsening or improvement of alopecia symptoms (18–20).

It is important to note that the following discussions are speculative hypotheses, as the exact mechanisms underlying the association between these drugs and alopecia remain unclear. Several hypotheses have been proposed to explain dupilumab-induced alopecia, ranging from disease exacerbation to therapeutic improvement (21, 22). One line of reasoning suggests that Th2 cell inhibition may inadvertently upregulate alternative immune pathways, particularly the Th1/Th17 axis, which is known to drive alopecia areata pathogenesis (23). In contrast, another perspective posits that dupilumab’s blockade of IL-4 signaling could dampen the production of pro-inflammatory mediators, potentially concurrently ameliorating both alopecia and AD manifestations (24). A third hypothesis proposes that IL-4 and IL-13 expression correlate positively with sebaceous gland function, and reduced activity of these cytokines may disrupt glandular development, leading to atrophy and secondary alopecia areata (25). Furthermore, studies have found that features of alopecia areata are associated with the activation of TH1, TH2, IL-23, and IL-9/TH9 cytokines (23). Future clinical trials of selective antagonists are needed to identify key pathogenic pathways and validate these hypotheses.

Beyond cutaneous manifestations, infectious complications represent another critical safety consideration. Infection-related AEs include eczema herpeticum (a cutaneous infection caused by HSV), conjunctivitis, and other ocular infections. Notably, eczema herpeticum is not listed in tralokinumab’s adverse reaction profile, despite a 0.3%–0.5% incidence in phase III trials—lower than the control group (10, 26). However, it is essential to note that eczema herpeticum may represent a manifestation or complication of severe AD rather than an accurate drug-related AE. Tralokinumab’s specific binding to IL-13 (without affecting IL-4) may contribute to this low risk, though the exact mechanism remains unclear. Proposed pathways include decreased expression of keratinocyte/tight junction proteins (e.g., claudins), impairing skin barrier integrity; reduced interferon/antimicrobial peptide production; and a predominantly Th2 immune response with abnormal regulatory T cell levels—all of which increase HSV susceptibility (27).

Ocular AEs constitute a significant safety concern associated with tralokinumab, with conjunctivitis being the most commonly reported event. Five randomized controlled trials demonstrated a 7.5% incidence of conjunctivitis in tralokinumab-treated patients compared to 3.2% in placebo-treated patients (11), and a meta-analysis confirmed a significantly elevated risk with tralokinumab (28). Notably, our study revealed that the proportion of tralokinumab-related conjunctivitis was considerably higher than that of dupilumab (2.4% vs. 1.4%, p=0.001). It should be emphasized that these percentages represent the proportion of reports, not the proportion of patients or the true incidence of these events. Risk factors for severe conjunctivitis include baseline AD severity, history of allergic conjunctivitis, atopic keratoconjunctivitis, and multiple atopic comorbidities (29). It is important to note that FAERS-based comparisons do not reflect accurate incidence rates, as they are subject to reporting biases and other limitations. Therefore, the higher reporting proportion of conjunctivitis with tralokinumab compared with dupilumab should be interpreted with caution and may not represent an actual difference in incidence.

The pathophysiology of biologic agent-related ocular AEs involves IL-4/IL-13-mediated disruption of conjunctival homeostasis. Monoclonal antibodies targeting IL-4/IL-13 (through IL-4Rα interaction) inhibit conjunctival goblet cell activation, decrease mucin secretion, and compromise tear film stability (30, 31). This cascade results in dysfunction of the mucosal epithelial barrier and subsequent ocular inflammation. Mechanistic explanations for these findings remain highly speculative and require further investigation. Specifically for tralokinumab, it is hypothesized that its selective inhibition of IL-13 may partially reduce this risk compared to dual IL-4/IL-13 inhibitors, based on current knowledge of IL-13’s role in ocular inflammation. However, this hypothesis is not directly supported by our FAERS-based finding that the reporting proportion of tralokinumab-related conjunctivitis was significantly higher than that of dupilumab. This discrepancy highlights the limitations of FAERS data for comparing the safety profiles of different biologic agents and the need for caution when interpreting such results. Further research is required to elucidate the relationship between tralokinumab’s selective IL-13 inhibition and the risk of ocular AEs, and to identify potential confounding factors that may account for the higher reporting proportion of conjunctivitis with tralokinumab compared to dupilumab.

Clinical management of tralokinumab-associated ocular AEs should follow a stepwise approach. Clinicians should assess symptom severity and initiate topical lubricants or antihistamine drops for mild cases. Persistent symptoms warrant referral to ophthalmology for slit-lamp examination and potential topical corticosteroid therapy. Close monitoring is critical, as untreated ocular inflammation may progress to keratitis or vision impairment. Other ocular AEs include eyelid irritation, eye pain, eye swelling, eye discharge, dry eye, ocular hyperemia, increased lacrimation, and blepharitis. Except for blepharitis, these reflect manifestations of conjunctivitis. Blepharitis, unlabeled for tralokinumab, has been reported with both biologics (8% incidence with dupilumab) (32). Studies suggest that by binding to IL-4Rα and thereby inhibiting IL-4 and IL-13, these monoclonal antibodies prevent conjunctival goblet cell activation, leading to hypoplasia and decreased mucin production, which, in turn, affects the stability of the tear film and the function of the mucosal epithelial barrier (33).

To our knowledge, there are currently no reports of visual impairment associated with tralokinumab, whereas several studies have reported visual impairment and blurred vision with dupilumab (34, 35). This study identified a signal for blurred vision AEs related to tralokinumab treatment. Therefore, it is essential to consult an ophthalmologist promptly when visual issues arise during treatment to minimize potential vision damage.

Our study also identified several AEs in the SOC of general disorders and administration site conditions, including injection site pain, injection site erythema, swelling, and rash at the injection site, as well as facial swelling. All these AEs have been documented in the medication instructions. Our research indicates that the risk of injection site reactions (including pain, swelling, and cyanosis) associated with dupilumab is significantly higher than that associated with tralokinumab (21.3% vs. 15.6%, p<0.001). Urticaria is an AE not listed on the drug label. At present, studies are reporting AE of urticaria caused by treatment with dupilumab (36). The exact pathogenesis of urticaria remains incompletely understood, but it typically involves the release of various mediators from mast cells, such as histamine, proteases, prostaglandins, leukotrienes, and cytokines (37).

Exfoliative dermatitis is a severe form of dermatitis that can be caused by a variety of factors, including underlying skin diseases, infections, and medications (38). Therefore, the occurrence of exfoliative dermatitis in patients treated with tralokinumab may be related to underlying disease severity rather than to the treatment itself. Further research is required to elucidate the relationship between tralokinumab treatment and the risk of exfoliative dermatitis. These findings emphasize the necessity for dermatologists to enhance patient monitoring and education throughout the treatment process.

Our results demonstrated that the median time to onset of tralokinumab-related AEs was 37 days, with most occurring within the first month of treatment. Only 14% of AEs occurred after 6 months of treatment, a clinically significant finding. This result suggests that the risk of tralokinumab-related AEs decreases significantly after six months of treatment, and clinicians may consider adjusting monitoring strategies accordingly. Timely identification of AEs by healthcare professionals is crucial for rapidly implementing preventive strategies and minimizing clinical impact on patients. However, no difference in the timing of conjunctivitis onset was observed between tralokinumab and dupilumab in this study; enhanced monitoring for specific AEs (e.g., conjunctivitis and keratitis) remains necessary in patients with AD treated with biologic agents to mitigate the impact of serious AEs.

## Conclusion

This study conducted a comprehensive pharmacovigilance analysis of tralokinumab-associated AEs using FAERS data, identifying potential safety signals not listed on the product label: eczema herpeticum, generalized exfoliative dermatitis, hair loss, skin exfoliation, and eyelid inflammation. Notably, eczema herpeticum, generalized exfoliative dermatitis, and skin exfoliation may reflect severe AD manifestations rather than true drug-related AEs, highlighting the risk of misclassifying disease vs. treatment-related events.

Comparative analysis with dupilumab revealed differential AE profiles: dupilumab had higher injection-site reaction reporting rates, while tralokinumab showed higher conjunctivitis and keratitis rates. These findings inform post-market safety monitoring and clinical decision-making by highlighting underrecognized tralokinumab-associated AEs requiring closer monitoring.

However, results should be interpreted cautiously due to limitations in the FAERS database (reporting biases and underreporting) and the observational study design. This study generates hypotheses that require validation through well-designed clinical trials to confirm safety signals, establish causal relationships, and characterize tralokinumab’s safety profile. Prospective trials with rigorous designs and larger sample sizes are needed to determine the accurate AE incidence rates, while mechanistic studies should elucidate underlying pathways and risk factors.

## Data Availability

The datasets presented in this study can be found in online repositories. The names of the repository/repositories and accession number(s) can be found in the article/**Supplementary Material**.
