# Supplementary material for: Investigating tralokinumab-related adverse events in treating atopic dermatitis: insights from the FAERS database
Source: Front Immunol. 2026 Feb 23;17:1769109. doi: 10.3389/fimmu.2026.1769109 (PMC12968180; doi:10.3389/fimmu.2026.1769109)
Supplement: Supplementary file 1 [file Table1.doc]

**Supplementary Table 1. Four major algorithms are** used for signal detection.

| Algorithms | Equation | Criteria |
| --- | --- | --- |
| ROR | ROR=ad/b/c | lower limit of 95% CI>1, a≥3 |
|  | 95%CI=eln(ROR)±1.96(1/a+1/b+1/c+1/d)^0.5 |  |
| MHRA | PRR=a(c+d)/c/(a+b) | PRR≥2, χ^2^≥4, a≥3 |
|  | χ^2^=[(ad-bc)^2](a+b+c+d)/[(a+b)(c+d)(a+c)(b+d)] |  |
| BCPNN | IC=log_2_a(a+b+c+d)(a+c)(a+b) | IC025>0 |
|  | 95%CI= E(IC) ± 2V(IC)^0.5 |  |
| MGPS | EBGM=a(a+b+c+d)/(a+c)/(a+b) | EBGM05>2 |
|  | 95%CI=eln(EBGM)±1.96(1/a+1/b+1/c+1/d)^0.5 |  |

Note: a, number of reports containing both the target drug and target adverse drug reaction; b, number of reports containing other adverse drug reactions of the target drug; c, number of reports containing the target adverse drug reaction of different medications; d, number of reports containing other medicines and other adverse drug reactions. IC, information component; IC025, the lower limit of 95% CI of the IC; E(IC), the IC expectations; V(IC), the variance of IC; EBGM, empirical Bayesian geometric mean; EBGM05, the lower limit of 95% CI of EBGM.
